# Supplementary material for: Estimating the trend of COVID-19 in Norway by combining multiple surveillance indicators
Source: PLoS One. 2025 Jan 30;20(1):e0317105. doi: 10.1371/journal.pone.0317105 (PMC11781655; doi:10.1371/journal.pone.0317105)
Supplement: Attachment — Submitted filename: ResponseToReviewers.pdf [file pone.0317105.s002.pdf]

Response to the reviewers

We thank the reviewers for insightful comments and provide a point-by-point answer below.

Reviewer 1

This paper is methodologically interesting, but I have some concerns about these points:

Reviewer Point P 1.1 — The authors characterize the Proportion Positive as an Incidence Indicator, but the definition they provide seems more like a Prevalence indicator, except they refer only to Positive tests in new cases. The definition in the paper states only positive tests divided by total tests, but as it has been seen later, one test can remain positive through different periods of time and ithe authors do not specify how they treat repeated tests.

Reply: The proportion positive PCR tests was classified as an incidence indicator as it is directly related to the number of new confirmed PCR cases by dividing by the total number of "test-events" per day. Since it is just a rescaling of new cases which is an incidence indicator, we consider proportion positive to also be an incidence indicator. Both the numerator, number of positive cases, and the test-event denominator are defined to remove the effect of the same person being tested multiple times. As described in line 93 (in the manuscript with track changes), test-events only consider one test per person in a 7 day period and for the number of positive tests we only consider a persons first positive test within a 60-day period, so repeated positive tests within this period are not included. We have updated the text in line 89-91 and a minor update in the "Proportion Positive" row in the table to clarify this aspect of positive tests.

Reviewer Point P 1.2 — Relating to Figure 4 trhe authors should make it clear why some correlations are not included (seemingly because there are no data for those indicators in the same period)

Reply: This is a good point, we have updated the caption for figure 4.

Reviewer Point P 1.3 — In the Results section the authors write: "When comparing the overall growth rate to the rates for 226 cases and hospitalisations we can see multiple times of disagreement, especially around 227 early 2022. The heterogeneity between the growth rates from the different indicators 228 varies significantly over the time with both early 2020 and early 2022 being examples of 229 periods with high heterogeneity". Nevertheless they conclude "that estimating and visualising growth rates from many 234 different surveillance indicators can provide a more complete picture of the spread of 235 COVID-19 than only considering laboratory confirmed cases or hospitalisations". As a public health specialist I would prefer to know which of the different indicators should be included in a robust model for estimating growth rates, as it seems that some of the indicators are not suitable for a robust model.

Reply: This is a good questions and while it would indeed have been useful to be able to determine which indicators are robust, our aim is instead to try to use all the available surveillance indicators to understand the underlying transmission process. Even less robust surveillance indicators can provide important information. During the pandemic all indicators had a changing relationship with the underlying infections, and that the best solution therefore is to consider all available indicators holistically as we show in figure 3 with all the growth rates. Using the meta-analysis model also allows us to combine the different growth rates into one when the heterogeneity is low, and gives us a signal that interpretation is

more difficult when heterogeneity is high. In these situations, one needs real epidemiological and public health insight into the disease processes, and the data collection and interpretation of each individual indicator to determine which indicator provides the best description of the underlying transmission. We have tried to clarify our aims in line 58-73, a minor edit in the abstract in line 5 and in the discussion in line 312-315.

Reviewer 2

Reviewer Point P 2.1 — Really I found a problem to understand the title of the article. It doesn't reflects the aim exactly. It should be more obvious.

Reply: We apologies if the aim was not described well enough. The aim is to use multiple surveillance indicators to provide a more comprehensive and robust estimate the underlying trend of new COVID-19 infections in Norway. We have tried to highlight this in line 58-73 (In the manuscript with track changes) and with a minor edit in the abstract in line 5. We hope that this makes it clear that the title corresponds to our aims in the article.

Reviewer Point P 2.2 — Also the estimate methods are not equivalent in time factor and area distribution which may cause some bias in comparison of data. Also you didn't mention other causes that could affect the growth rate at the same time.

Reply: We completely agree that there are differences in geographical area, age of participants and many other characteristics between the different surveillance indicators. This is discussed in line 302-310. As we commented on above, the goal of the paper is not to compare and contrast the different indicators, but to extract useful estimates of the overall trend in new infections. The high correlation between the indicators clearly show that even if there are differences between the different indicators, they also all provide information about the trend in the underlying transmission process. We have clarified this further in the lines 312-315.

Reviewer Point P 2.3 — Although you said that the ethical approval is not needed. But I think there should be.

Reply: The data we have used contain no identifiable data and only consist of the aggregated number of events (cases/admission/deaths/etc) on a national level. The data has been collected and prepared for surveillance purposes and have already been published in weekly reports by the Norwegian Public Health Institute(https://www.fhi.no/publ/statusrapporter/luftveisinfeksjoner/) and in part in many other publications(i.e https://github.com/folkehelseinstituttet/surveillance_data). Since this is just a reanalysis of data already collected, used and published for public health surveillance purposes, no additional ethical approval is needed. We have clarified this in line 208.
